# Efficacy and Safety of *Lactobacillus acidophilus* LA85 in Preventing Antibiotic‐Associated Diarrhea: A Randomized, Placebo‐Controlled Study

**DOI:** 10.1002/fsn3.70490

**Published:** 2025-06-20

**Authors:** Jianguo Zhu, Yukun Sun, Yao Dong, Yunjiao Zhao, Zhonghui Gai, Shuguang Fang

**Affiliations:** ^1^ Wecare Probiotic Research and Development Center (WPC) Wecare Probiotics Co., Ltd Suzhou China

**Keywords:** amoxicillin, antibiotic‐associated diarrhea, *Lactobacillus acidophilus*, probiotic, randomized controlled trial

## Abstract

Antibiotic‐associated diarrhea (AAD) is a common clinical complication resulting from antibiotic‐induced gut microbiota dysbiosis. *Lactobacillus acidophilus* LA85, a probiotic strain illustrated with in vitro antimicrobial and immunomodulatory properties, may offer a preventive approach against AAD. However, clinical evidence on its efficacy remains limited. This randomized, double‐blind, placebo‐controlled trial evaluated the efficacy and safety of *L. acidophilus* LA85 in preventing amoxicillin‐associated diarrhea. A total of 82 adult participants receiving amoxicillin treatment were randomized to receive either LA85 (2 × 10^9^ CFU/day) or placebo for 14 days. The primary outcomes included AAD incidence, diarrhea duration, and stool consistency, while secondary outcomes assessed gastrointestinal quality of life and safety. LA85 supplementation was associated with a trend toward a reduction in the incidence of AAD; however, this difference did not reach statistical significance. Nonetheless, LA85 notably shortened the duration of diarrhea episodes compared to placebo (*p* = 0.072), suggesting a clinically meaningful improvement. Participants receiving LA85 exhibited less variability in stool consistency scores, assessed by the Bristol stool form scale (BSFS), maintaining scores consistently around 3.5. In contrast, placebo recipients had greater fluctuations between 3.5 and 4.0, indicating less stable stool consistency during antibiotic treatment. Importantly, exploratory subgroup analysis revealed that in younger participants (< 53 years age), LA85 supplementation significantly reduced the incidence of AAD (*p* = 0.008) and effectively eliminated persistent diarrhea episodes. Gastrointestinal quality of life scores improved significantly in the probiotic group (*p* < 0.05). No serious adverse events were reported, supporting the safety of LA85. While these findings support the clinical application of LA85 for preventing AAD, further large‐scale trials incorporating microbiome analysis and longer follow‐up periods are necessary to confirm its long‐term benefits and generalizability.

**Trial Registration:** This clinical trial (ClinicalTrials.gov Identifier: NCT05974657) was registered on August 3, 2023

## Graphical abstract

*Lactobacillus acidophilus* LA85 supplementation showed potential in reducing antibiotic‐associated diarrhea (AAD) incidence and significantly shortening diarrhea duration, particularly in younger and highly adherent participants. It improved stool consistency and gastrointestinal quality of life, with no serious adverse events, supporting its safety and clinical applicability.

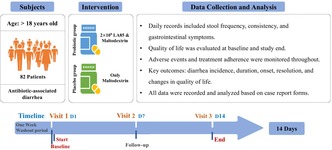

## 1 Introduction

Antibiotic‐associated diarrhea (AAD), defined as unexplained diarrhea linked to antibiotic administration, is a common clinical complication with incidence rates varying from 5% to 35%, influenced by antibiotic type, dosage, treatment duration, and patient susceptibility (Saviano et al. 2024; Xu et al. 2024). AAD primarily results from antibiotic‐induced dysbiosis, characterized by disruptions in gut microbiota composition, facilitating the proliferation of pathogenic or opportunistic microorganisms (Lai et al. 2024). Clinically, AAD manifests as abdominal discomfort, bloating, and watery stools, significantly compromising patients' quality of life and potentially reducing adherence to antibiotic therapies (Gallo et al. 2024; Obeagu and Obeagu 2024). Among broad‐spectrum antibiotics, amoxicillin is notably associated with gastrointestinal disturbances due to its extensive impact on commensal gut flora, underscoring the clinical need for effective preventive strategies (Fishbein et al. 2023; Lathakumari et al. 2024). Current preventive strategies for AAD primarily include symptomatic management, dietary interventions, and probiotic supplementation. Among these, probiotics, live microorganisms that confer health benefits when administered in adequate amounts (Sanders 2003), have garnered significant research interest due to their ability to directly address gut microbiota dysbiosis induced by antibiotic treatments (Maftei et al. 2024). Probiotics exert preventive effects against AAD through various mechanisms, including competitive exclusion of pathogenic microorganisms, reinforcement of intestinal barrier integrity, modulation of gut microbiota composition, and immunoregulatory actions (Karimi and Hosseinzadeh 2024; Kaur and Ali 2022). Despite growing support for probiotics, clinical trials have yielded inconsistent outcomes, likely due to substantial variability in the efficacy of individual probiotic strains. This strain‐specificity underscores the necessity of rigorous evaluation for each probiotic candidate, as efficacy results for one strain may not translate broadly across a given species.

*Lactobacillus acidophilus* has emerged as a probiotic candidate for AAD prevention, with certain strains demonstrating efficacy through mechanisms such as pathogen exclusion, microbiota modulation, and gut barrier enhancement (Ma et al. 2023; Wen et al. 2023). However, clinical evidence on strain‐specific benefits remains inconsistent, highlighting that probiotic efficacy cannot be generalized across the entire species. *Lactobacillus acidophilus* LA85, a probiotic strain patented by Wecare Probiotics Co. Ltd., has exhibited promising in vitro characteristics, including robust resistance to gastrointestinal conditions, strong adherence to intestinal epithelia, pathogen antagonism, and demonstrated safety (Qi et al. 2024; Wen et al. 2023). Additionally, previous preclinical studies have showed significant efficacy of LA85 in improving intestinal inflammation and barrier function in mouse models of ulcerative colitis (UC), providing further support for its beneficial potential (Han et al. 2023). Nonetheless, rigorous clinical evidence specifically evaluating its effectiveness in preventing AAD remains limited. Therefore, this study aims to evaluate the clinical efficacy and safety of *L. acidophilus* LA85 supplementation in preventing amoxicillin‐associated diarrhea through a randomized, double‐blind, placebo‐controlled clinical trial. We hypothesize that LA85 supplementation will significantly reduce AAD incidence, shorten diarrhea duration, improve stool consistency, and enhance gastrointestinal‐related quality of life, thereby offering robust evidence for its clinical application.

## 2 Materials and Methods

### 2.1 Study Design and Ethical Considerations

A randomized, double‐blind, placebo‐controlled clinical trial (ClinicalTrials.gov Identifier: NCT05974657) was conducted between September 2021 and July 2022 in two private dental clinics in Barcelona province (Badalona and Castelldefels) as part of routine clinical practice. The study protocol was approved by the Clinical Research Ethical Committee Parc de Salut Mar (Barcelona, Spain) (study code: MTH_WEC0220) and adhered to the Declaration of Helsinki and its subsequent revisions. Written informed consent was obtained from all participants prior to enrollment.

### 2.2 Inclusion and Exclusion Criteria

Eligible participants were adults aged 18 years or older who were prescribed amoxicillin therapy (750 mg twice daily for 7 consecutive days) and were capable of understanding and complying with all study procedures. Exclusion criteria were established to minimize potential confounding factors and ensure participant safety. Participants were excluded if they had any conditions known to alter gastrointestinal function or gut microbiota composition, including diabetes mellitus, pregnancy, or lactation. These conditions may affect intestinal motility, microbial community stability, and mucosal immune responses, which could influence probiotic efficacy or safety assessments. Additionally, individuals currently taking medications commonly associated with diarrhea were excluded. These included, but were not limited to, laxatives, magnesium‐containing antacids, chemotherapeutic agents, proton‐pump inhibitors (PPIs), H2‐receptor antagonists, mycophenolate, non‐steroidal anti‐inflammatory drugs (NSAIDs), metformin, and opioids. Participants receiving other pharmacologic agents that may interfere with bowel motility or microbial balance, such as anti‐diarrheal drugs, tricyclic antidepressants, monoamine oxidase (MAO) inhibitors, antiepileptics, antihistamines, antipsychotics, verapamil, and sympathomimetic drugs, were also excluded. During screening, study physicians conducted individualized assessments to identify additional medications or comorbidities that could influence gastrointestinal outcomes. Furthermore, individuals who had recently initiated or planned to implement substantial dietary modifications during the study period were excluded. This included transitions to ketogenic, vegan, or high‐fiber therapeutic diets, which could independently impact bowel habits or microbiota composition. Other exclusion criteria included known allergies or intolerances to components of the investigational product, and a history of alcohol or substance abuse.

### 2.3 Intervention, Randomization, and Blinding Procedures

Participants were randomly assigned in a 1:1 ratio to receive either a probiotic formulation containing *Lactobacillus acidophilus* LA85 (2 × 10^9^ CFU) with 20 mg of maltodextrin (total 400 mg) or a placebo containing 400 mg of maltodextrin. The probiotic strain *L. acidophilus* LA85 was provided by Wecare Probiotics Co. Ltd. (Jiangsu, China) using a patented fermentation, enrichment, and freeze‐drying process to maintain viability. Both formulations were encapsulated in hydroxypropyl cellulose capsules and administered once daily for 14 days. Participants who were consuming probiotics at the time of enrollment underwent a one‐week washout period before starting the study intervention. Randomization was performed using a permuted block design (block size = 4), with the randomization sequence generated in R software. Allocation concealment was ensured through sequentially numbered, opaque, sealed envelopes prepared by an independent researcher not involved in the study. To implement the double‐blind design, two levels of blinding were employed: Level 1 (product blinding): The test and placebo capsules were made visually indistinguishable, with identical appearance, packaging, labeling, and instructions. Level 2 (code blinding): Each package was labeled with a code corresponding to the randomization sequence. The entire blinding and packaging process was completed and documented by personnel from the independent third‐party commissioning unit, who were not involved in clinical observation or outcome assessment. Throughout the study, participants, investigators, outcome assessors, and data analysts remained blinded to group assignments.

### 2.4 Study Procedures

At Visit 1 (Baseline, Day 0), participants received a 14‐day supply of study capsules, with the first dose administered at recruitment. They were provided with a daily diary to record stool consistency using the Bristol Stool Scale (ranging from type 1 = hard lumps to type 7 = watery stools), stool frequency, presence of mucus or blood, and gastrointestinal symptoms such as abdominal pain, weakness, and loss of appetite (Blake et al. 2016; Chumpitazi et al. 2016). Demographic and clinical characteristics, including dietary habits based on WHO criteria (https://www.who.int/news‐room/fact‐sheets/detail/healthy‐diet), were documented. Additionally, participants completed the Spanish‐validated Gastrointestinal Quality of Life Index (GIQLI) (Eypasch et al. 1995), a 36‐item questionnaire assessing gastrointestinal symptoms, physical status, emotions, and social dysfunction, with scores ranging from 0 to 144, where higher scores indicate better quality of life (Quintana et al. 2001). At Visit 2 (Day 7) and Visit 3 (Day 14), investigators reviewed the patient diaries to assess symptom progression and monitored the occurrence of adverse events (AEs) and treatment compliance. At Visit 3, participants completed the GIQLI questionnaire again to evaluate changes in quality of life. Participants were also required to return unused medication to verify compliance. All collected data were recorded in case report forms (CRFs) for further analysis.

### 2.5 Outcomes and Definitions

The primary efficacy outcomes included the incidence of diarrhea, defined as ≥ 2 stools per day with a Bristol Stool Scale score of ≥ 5 occurring for at least two consecutive days, as well as the duration of diarrhea (total days in a diarrheal state) and the time to diarrhea onset (days from study initiation to the first diarrheal event). Secondary efficacy outcomes included the time to resolution of diarrhea, the percentage of patients recovering from diarrhea, changes in Bristol stool score, and quality of life improvements based on gastrointestinal quality of life (GIQLI) scores. The primary safety outcome was the prevalence of adverse events (AEs) in both treatment groups. The sample size was calculated to include 82 participants (41 per group), providing 80% power to detect significant differences in diarrhea incidence and time to onset with a 95% confidence level (Kopacz and Phadtare 2022).

### 2.6 Statistical Analysis

Descriptive statistics were performed for continuous variables, including the mean, median, standard deviation, and range, while categorical variables were summarized as frequency counts and percentages. The Kolmogorov–Smirnov test was used to assess normality. Within‐group comparisons across different time points (T0, T7, and T14) were conducted using the Wilcoxon signed‐rank test for paired non‐parametric data, while between‐group comparisons were performed using the Mann–Whitney *U* test for unpaired non‐parametric data. A two‐sided *p*‐value < 0.05 was considered statistically significant for all analyses.

## 3 Results

### 3.1 Baseline Characteristics of Participants

A total of 82 participants were randomized into the placebo group (*n* = 41) and the probiotic group (LA85, *n* = 41), with demographic and clinical characteristics well‐balanced between groups (Table 1). Gender distribution was identical in both groups, with 56.1% female and 43.9% male participants (*p* = 1.000), indicating that gender is not a confounding factor. The mean age of participants was 47.3 years, with the placebo group being slightly younger (44.9 ± 11.58 years) than the probiotic group (50.9 ± 9.89 years, *p* = 0.035). While statistically significant, this six‐year age difference is unlikely to have strong clinical implications and will be further analyzed in relation to treatment outcomes. All enrolled participants were over 18 years age. For exploratory subgroup analysis, we used 53 years as an approximate midpoint of the observed age distribution to categorize participants into relatively younger and older groups, in order to investigate whether age may influence the intervention response. Nutritional status and metabolic indicators were comparable between groups, with mean body weight slightly lower in the probiotic group (69.6 kg vs. 73.7 kg, *p* = 0.244) and BMI values similar (24.57 vs. 25.49, *p* = 0.568), suggesting no substantial differences in baseline health conditions. Dietary habits were also well‐matched, with 95.1% of participants adhering to a specific diet, and a slightly higher proportion in the probiotic group (97.6% vs. 92.7%, *p* = 0.616), though this difference was not significant. The prevalence of irritable bowel syndrome (IBS) was minimal, with only one participant (1.22%) diagnosed with IBS in the placebo group and none in the probiotic group (*p* = 1.000), making it unlikely to influence study outcomes. Treatment compliance was high in both groups, with 76 participants (92.7%) classified as always complied (i.e., took the study product daily without missing any doses), and 6 participants (7.3%) classified as “almost always complied” (i.e., missed no more than 1–2 doses, with an adherence rate ≥ 85%). The distribution of adherence was similar between groups (*p* = 0.675). These data support the reliability of the study outcomes. Overall, the baseline characteristics demonstrate a well‐balanced population, apart from a minor age difference, which will be considered in subsequent analyses to evaluate its impact on treatment effects.

**TABLE 1:** Baseline characteristics of patients.

	All (*n* = 82)	Placebo (*n* = 41)	LA85 (*n* = 41)	*p*
Gender				1.000
Female	46 (56.1%)	23 (56.1%)	23 (56.1%)	
Male	36 (43.9%)	18 (43.9%)	18 (43.9%)	
Age	47.3 ± 11.12	44.9 ± 11.58	50.9 ± 9.89	0.035
Height (cm)	168.7 ± 9.51	169.7 ± 9.91	167.7 ± 9.11	0.411
Weight (kg)	71.6 ± 16.79	73.7 ± 17.17	69.6 ± 16.35	0.244
BMI	25.03 ± 4.77	25.49 ± 5.10	24.57 ± 4.44	0.568
Diet				0.616
Yes	78 (95.1%)	38 (92.7%)	40 (97.6%)	
No	4 (4.88%)	3 (7.32%)	1 (2.44%)	
IBS	1 (1.22%)	1 (2.44%)	0 (0)	1.000
Adherence to treatment				0.675
Always complied	76 (92.7%)	39 (95.1%)	37 (90.2%)	
Almost always complied	6 (7.32%)	2 (4.88%)	4 (9.76%)	

Abbreviations: IBS, irritable bowel syndrome; LA85, *Lactobacillus acidophilus* LA85.

### 3.2 Effects of LA85 on Diarrhea Incidence and Duration

The impact of LA85 on AAD was assessed by comparing the duration, incidence, and onset of diarrhea episodes between the placebo and LA85 groups (Table 2). The mean duration of diarrheal episodes was shorter in the probiotic group (0.0976 days, ~2.34 h) than in the placebo group (0.415 days, ~9.96 h), with a *p*‐value of 0.072, indicating a trend toward significance. Although this difference did not reach statistical significance (*p* < 0.05), the 77% reduction in diarrhea duration suggests a potential clinical benefit that warrants further investigation. The incidence of diarrhea was also lower in the probiotic group, with only 4.9% (2/41) of participants experiencing diarrhea, compared to 14.6% (6/41) in the placebo group (*p* = 0.0615), again showing a trend toward a preventive effect of LA85. However, the time to onset of diarrhea was similar between groups (placebo: 4.00 ± 2.90 days vs. probiotic: 4.50 ± 0.71 days, *p* = 0.3042), suggesting that while LA85 may help reduce the severity and duration of diarrhea, it does not significantly alter the timing of symptom onset.

**TABLE 2:** Effects of *Lactobacillus acidophilus* LA85 on diarrhea outcomes in the overall cohort and subgroup analyses.

Population group	Group	Diarrhea duration (days)	% With diarrhea	Days to onset	*p* (duration)	*p* (% diarrhea)
Overall (*n* = 82)	Placebo (*n* = 41)	0.415 (1.61)	14.6% (6)	4.00 (2.90)	0.072	0.0615
	LA85 (*n* = 41)	0.0976 (0.49)	4.9% (2)	4.50 (0.71)		
Always complied (*n* = 76)	Placebo (*n* = 39)	0.227 (0.612)	13.6% (3)	1 (0.000)	0.008	0.241
	LA85 (*n* = 37)	0.000 (0)	0.0% (0)	—		
Age < 53 years old (*n* = 53)	Placebo (*n* = 29)	0.517 (1.88)	17.2% (5)	4.4 (3.05)	0.018	0.048
	LA85 (*n* = 24)	0.000 (0)	0.0% (0)	—		

*Note:* Effects of *Lactobacillus acidophilus* LA85 on diarrhea outcomes in the overall cohort and subgroups. Data are shown as mean ± SD or number (%). Subgroups include participants with full compliance and those under 53 years. Outcomes include diarrhea duration and time to onset.

### 3.3 Diarrheal Outcomes in Subjects With Full Compliance

To assess the impact of LA85 in subjects with high adherence to the study protocol, a subgroup analysis was conducted among participants who consistently complied with treatment (Table 2). The duration of diarrheal episodes was significantly shorter in the probiotic group (0.000 days) compared to the placebo group (0.227 ± 0.612 days, *p* = 0.008), indicating that none of the participants in the LA85 group experienced prolonged diarrhea. Additionally, while the incidence of diarrhea was lower in the probiotic group (0.0% vs. 13.6% in the placebo group), the difference did not reach statistical significance (*p* = 0.241), likely due to the small number of events observed. The time to diarrhea onset was recorded in the placebo group (mean: 1 day), whereas no cases of diarrhea were reported in the probiotic group. These findings suggest that strict adherence to LA85 supplementation may provide enhanced protective effects against AAD, effectively preventing its occurrence and completely eliminating prolonged diarrheal episodes. However, the non‐significant difference in incidence underscores the need for further studies with larger sample sizes to validate the observed preventive effects.

### 3.4 Diarrheal Outcomes in Participants Under 53 Years of Age

A subgroup analysis was conducted to evaluate the impact of *Lactobacillus acidophilus* LA85 on AAD in participants under 53 years old. The mean duration of diarrhea was 0.517 ± 1.88 days in the placebo group, whereas no cases of diarrhea were observed in the probiotic group (0.000 days, *p* = 0.018). This significant reduction suggests that LA85 effectively prevents prolonged diarrhea episodes in younger individuals. Additionally, the incidence of diarrhea was significantly lower in the probiotic group (0.0% vs. 17.2% in the placebo group, *p* = 0.048), further supporting the preventive role of LA85 in this population. The time to diarrhea onset in the placebo group was 4.4 ± 3.05 days, but since no diarrhea cases were reported in the probiotic group, this variable could not be analyzed. These findings indicate that LA85 not only reduces diarrhea duration but may also completely prevent its occurrence in younger individuals, suggesting a potentially stronger protective effect in this demographic. The underlying mechanisms for this age‐related difference could be attributed to greater gut microbiota plasticity in younger individuals, allowing probiotics to colonize and exert beneficial effects more efficiently, or differences in immune response, with younger participants exhibiting a more favorable immunomodulatory reaction to probiotic supplementation.

### 3.5 Effects of LA85 on GIQLI Scores

The impact of LA85 on gastrointestinal‐related quality of life was assessed using the GIQLI (Table 3), which evaluates various domains including gastrointestinal symptoms, emotional well‐being, physical health, and social functioning. Compared to the placebo group, the probiotic group showed significant improvements in several key aspects of quality of life over the 14‐day intervention period. Notably, the enjoyment of eating improved significantly in the probiotic group (*p* = 0.043), suggesting that LA85 may contribute to better digestive comfort and meal satisfaction. Additionally, participants in the probiotic group reported significantly less perceived physical deterioration compared to the placebo group (*p* = 0.003), indicating a potential benefit in maintaining overall physical well‐being. Furthermore, the ability to perform daily activities, including work, household tasks, and studies, improved significantly in the probiotic group (*p* = 0.033), highlighting the potential role of LA85 in reducing gastrointestinal discomfort that interferes with daily life. Although differences in other GIQLI components, including abdominal pain, bloating, and stool‐related symptoms, did not reach statistical significance, the probiotic group exhibited a general trend toward improvement in gastrointestinal well‐being compared to placebo. These findings suggest that LA85 supplementation may enhance overall quality of life by reducing digestive discomfort and improving functional outcomes, reinforcing its potential role in the management of antibiotic‐associated gastrointestinal disturbances.

**TABLE 3:** Results of the difference between T0 and T14 of the GIQLI questionnaire.

Item	Question	Placebo (*n* = 41)	LA85 (*n* = 41)	*p*
1	Have you felt stomach or stomach pain?	0.07 (0.85)	−0.15 (0.73)	0.379
2	Have you felt a feeling of abdominal fullness?	−0.10 (0.97)	−0.07 (1.01)	0.929
3	Have you felt bloating?	0.02 (1.01)	−0.29 (1.27)	0.473
4	Have you felt escape from winds?	0.05 (0.97)	0.02 (0.85)	0.866
5	Have you felt strong belching?	−0.17 (0.89)	0.05 (0.74)	0.194
6	Have you felt flashy noises in your stomach or gut?	−0.07 (0.82)	−0.12 (0.93)	0.956
7	Have you felt the need to do belly very often?	−0.15 (0.85)	0.15 (0.69)	0.090
8	Have you enjoyed or felt pleasure eating?	−0.41 (1.14)	0.20 (1.21)	**0.043**
9	Have you given up foods you like due to health?	0.00 (0.99)	−0.10 (1.17)	0.566
10	How have you coped with daily stress?	0.12 (1.03)	−0.12 (0.87)	0.173
11	Have you felt sad or depressed?	0.12 (0.78)	−0.10 (1.14)	0.636
12	Have you felt nervous or afraid?	0.07 (0.98)	−0.07 (1.03)	0.755
13	Have you been satisfied with your life overall?	0.34 (1.32)	0.07 (1.10)	0.362
14	Have you been frustrated?	0.00 (0.81)	−0.17 (0.77)	0.612
15	Have you felt tired or fatigued?	0.12 (1.00)	−0.02 (0.88)	0.497
16	Have you felt unwell?	−0.07 (0.61)	−0.02 (0.72)	0.604
17	Have you woken up at night?	0.12 (1.55)	0.29 (1.12)	0.597
18	Has your health caused annoying changes in appearance?	−0.03 (0.73)	−0.10 (1.06)	0.780
19	Has your vitality worsened due to health?	−0.07 (0.85)	−0.20 (1.45)	0.868
20	Have you lost your stamina due to health?	0.27 (1.05)	−0.29 (1.29)	0.078
21	Have you felt diminished in your physical form?	0.51 (1.19)	−0.32 (1.27)	**0.003**
22	Have you felt upset by medical treatment?	−0.17 (1.15)	0.03 (0.85)	0.650
23	Have you been able to carry out daily activities?	−0.46 (1.14)	0.02 (1.08)	**0.033**
24	Have you participated in recreational activities?	−0.02 (1.42)	−0.05 (1.09)	0.967
25	Have relationships with close people been altered?	0.07 (0.85)	−0.41 (1.26)	0.217
26	Has your sex life been impaired due to health?	0.26 (0.79)	−0.25 (1.16)	0.130
27	Have you felt regurgitation?	−0.02 (0.79)	0.05 (0.84)	0.811
28	Have you felt discomfort from eating slowly?	0.12 (0.75)	−0.07 (0.88)	0.409
29	Have you had trouble swallowing food?	−0.07 (0.65)	−0.02 (0.57)	0.863
30	Have you felt the need to do belly urgently?	0.00 (0.74)	−0.46 (1.40)	0.169
31	Have you had diarrhea?	0.02 (1.08)	−0.29 (0.84)	0.132
32	Have you had constipation?	−0.28 (1.06)	−0.10 (1.34)	0.505
33	Have you had nausea?	−0.02 (0.76)	−0.15 (0.48)	0.383
34	Have you had blood in your stool?	0.10 (0.62)	−0.02 (0.42)	0.145
35	Have you had heartburn?	0.20 (0.93)	−0.10 (0.77)	0.261
36	Have you had trouble holding your stool?	0.00 (0.61)	−0.39 (1.22)	0.237

*Note:* Changes in individual item scores of the Gastrointestinal Quality of Life Index (GIQLI) from baseline (T0) to Day 14 (T14) in the placebo and LA85 groups. Values are expressed as mean change ± standard deviation. Positive values indicate symptom improvement for positively worded items and symptom worsening for negatively worded items. Statistically significant differences between groups are highlighted in bold (*p* < 0.05).

Abbreviations: GIQLI, Gastrointestinal Quality of Life Index; LA85, *Lactobacillus acidophilus* LA85.

### 3.6 Effects of LA85 on Stool Consistency Scores and Diarrhea Incidence

**FIGURE 1:**
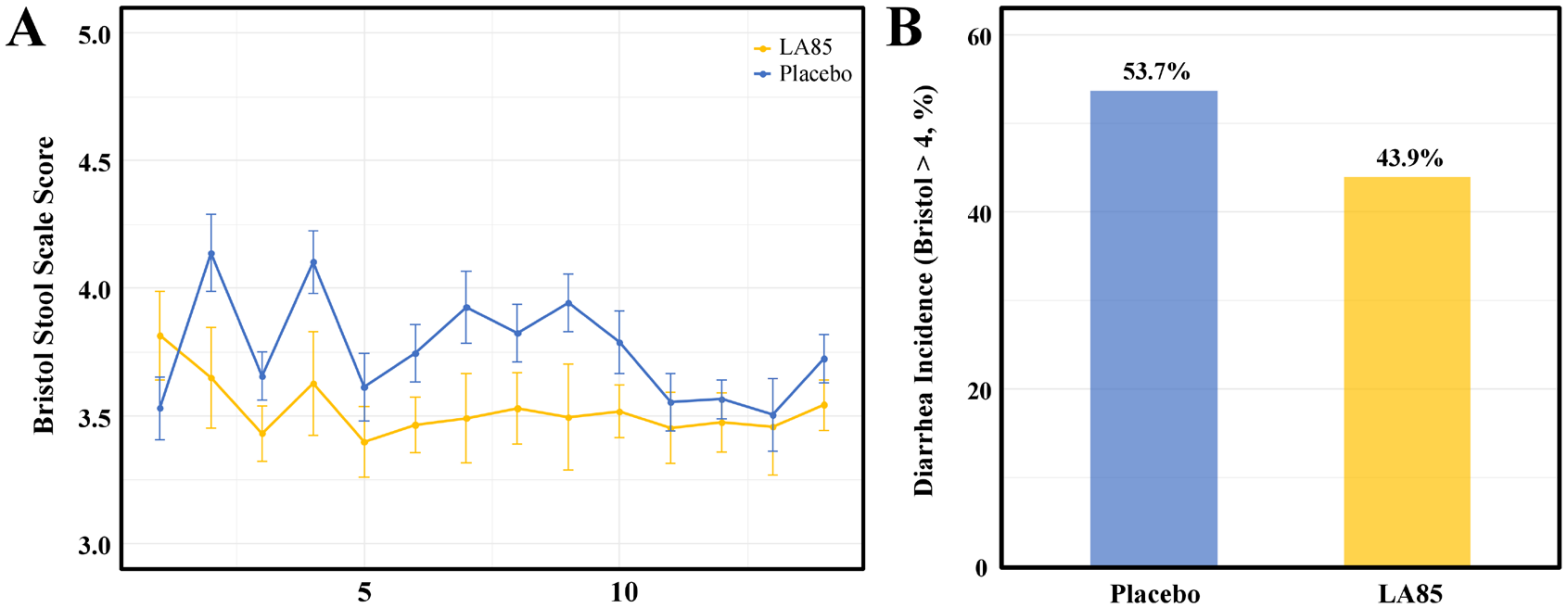
Effects of *Lactobacillus acidophilus* LA85 supplementation on stool consistency and diarrhea incidence. (A) Bristol Stool Scale scores over the intervention period in the LA85 and placebo groups. (B) Diarrhea incidence (defined as Bristol Stool Scale ≥ 4) in the placebo and LA85 groups.

Figure 1 illustrates differences between the placebo and LA85 groups in terms of stool consistency scores and diarrhea incidence. As shown in Figure 1A, throughout the intervention period, the LA85 group consistently exhibited lower Bristol stool scale scores compared to the placebo group, indicating more stable stool consistency and less severe diarrhea symptoms. Specifically, although both groups showed similar trends from day 1 to the end of the trial, the LA85 group's scores remained consistently lower, suggesting a potential beneficial effect of LA85 supplementation on stool consistency. Figure 1B demonstrates that diarrhea incidence (defined as a Bristol score ≥ 4) in the LA85 group was 43.8%, which was lower than the placebo group's 53.7%; however, this difference did not reach statistical significance.

## 4 Discussion

This randomized, double‐blind, placebo‐controlled clinical trial evaluated the clinical efficacy and safety of *Lactobacillus acidophilus* LA85 for preventing AAD. Results indicated that the LA85 group exhibited a reduced incidence of diarrhea compared to the placebo group. Although this difference did not reach statistical significance, it demonstrated a clinically meaningful beneficial trend. Importantly, diarrhea duration was notably shorter in the LA85 group, suggesting meaningful clinical improvements. Moreover, subgroup analysis among participants with high compliance showed that LA85 supplementation significantly decreased the incidence of AAD and completely prevented persistent diarrhea, highlighting a pronounced protective effect in this subgroup. Throughout the intervention period, the LA85 group consistently showed better Bristol Stool Scale scores compared to the placebo group, further supporting the probiotic's role in stabilizing gut function. Additionally, the observed improvements in gastrointestinal quality of life suggest LA85 has substantial potential to enhance patient well‐being, emphasizing its clinical relevance and suitability for broader clinical application.

A mechanistic exploration suggests that the preventive effects of LA85 against AAD may involve multiple pathways. Firstly, LA85 exhibits significant antimicrobial activity (Han et al. 2023; Xue et al. 2022), likely through competitive exclusion, nutrient competition, and the secretion of antimicrobial compounds such as lactic acid, hydrogen peroxide, and bacteriocins, which directly inhibit the overgrowth of pathogenic bacteria, including *Clostridium perfringens* , *Salmonella* spp., and enteropathogenic *Escherichia coli* (Vinayamohan et al. 2024). Previous studies have demonstrated that *Lactobacillus* strains can lower intestinal pH, inhibit pathogen adhesion, and suppress toxin production, thereby reducing the incidence of antibiotic‐associated diarrhea (Goodman et al. 2021; Pan et al. 2022; Yang et al. 2024). Additionally, probiotic colonization may further contribute to AAD prevention by competitively excluding antibiotic‐resistant pathogens and reducing their intestinal colonization (Gorreja and Walker 2022; Helmy et al. 2023). Our findings provide additional clinical insights, particularly regarding the age‐dependent efficacy of LA85. The subgroup analysis indicated that LA85 demonstrated a more pronounced protective effect in individuals under 53 years of age, which may be attributed to differences in gut microbiota composition, immune function, and antibiotic metabolism across age groups (Bosco and Noti 2021; Ling et al. 2022). Previous research has shown that younger individuals tend to have higher gut microbiota diversity and resilience following antibiotic exposure, potentially enhancing the ability of probiotics to restore microbial homeostasis (Bosco and Noti 2021; Shi et al. 2023). Moreover, aging is associated with increased gut permeability and a decline in mucosal immune function, which may partially explain the diminished probiotic efficacy observed in older individuals (Ling et al. 2022). These findings suggest that the clinical benefits of LA85 may be more pronounced in younger populations due to their enhanced microbiota plasticity and immune responsiveness, which warrant further investigation. Compared to other studies, our results further support the clinical benefits of LA85 in maintaining intestinal homeostasis. Although this study did not perform gut microbiota sequencing, the observed improvements in diarrhea symptoms and stool consistency suggest that LA85 may exert its protective effects through microbiota modulation. Future studies incorporating metagenomic and metabolomic analyses are necessary to elucidate the precise mechanisms underlying LA85's age‐dependent effects and its broader implications for AAD prevention in different populations (Puig‐Castellví et al. 2023).

The stability of the gut microbiota is a critical determinant of host health (Dzierozynski et al. 2023), and antibiotic use disrupts this balance by reducing the abundance of beneficial bacteria (Duan et al. 2022), such as *Lactobacillus* and *Bifidobacterium* species, while facilitating the overgrowth of opportunistic pathogens, including *Proteobacteria* and *Clostridioides difficile*, thereby increasing the risk of AAD (Dey and Ray Chaudhuri 2023; Tewari and Dey 2024). This dysbiosis is characterized by a loss of microbial diversity, altered metabolic activity, and increased gut permeability, all of which contribute to diarrhea and gastrointestinal discomfort (Singh et al. 2021; Stolfi et al. 2022). Restoration of microbial homeostasis is thus a key therapeutic target for preventing and managing AAD. Previous studies have demonstrated that *Lactobacillus acidophilus* can actively promote the proliferation of commensal bacteria, such as *Bifidobacterium*, which play a crucial role in maintaining gut health by producing metabolites such as short‐chain fatty acids and antimicrobial peptides (Guo et al. 2025). Additionally, *L. acidophilus* has been reported to suppress the expansion of antibiotic‐induced *Proteobacteria*, a phylum strongly associated with gut inflammation and dysbiosis (Duan et al. 2022). This is particularly relevant in the context of AAD, where an overrepresentation of *Proteobacteria* has been linked to increased intestinal permeability and immune dysregulation, exacerbating symptoms (Napolitano et al. 2023). Furthermore, metagenomic analyses have revealed that probiotic supplementation can enhance the production of SCFAs, including butyrate, acetate, and propionate, which serve as key energy sources for colonic epithelial cells, strengthen gut barrier integrity, and exhibit anti‐inflammatory properties by modulating cytokine release and immune cell activation (Liu et al. 2021; Salvi and Cowles 2021). These findings align with the results of our study, which highlighted that LA85 supplementation contributed to improved stool consistency and a reduction in diarrhea duration. These effects may be mediated through its ability to modulate gut microbiota composition, suppress pathogen overgrowth, and enhance SCFA production, thereby restoring gut homeostasis (Caballero‐Flores et al. 2023; Khan et al. 2021). However, given that our study did not include direct microbiome analysis, future investigations utilizing high‐throughput sequencing and metabolomic profiling will be necessary to elucidate the specific microbial shifts and functional changes associated with LA85 supplementation. Understanding these mechanisms in greater detail will provide more robust evidence for the role of *L. acidophilus* in AAD prevention and inform precision probiotic interventions for individuals at risk of antibiotic‐associated dysbiosis.

Restoration of intestinal mucosal barrier function is a crucial factor in preventing AAD. Tight junction proteins, such as occludin and zonulin, play a pivotal role in maintaining intestinal epithelial integrity by regulating paracellular permeability and preventing pathogen translocation (Serek and Oleksy‐Wawrzyniak 2021). Antibiotic use is often associated with a decline in the expression of these proteins, leading to compromised barrier function, increased intestinal permeability, and subsequent endotoxemia and systemic inflammation (Mohammad and Thiemermann 2021). This disruption is particularly concerning as it facilitates the translocation of bacterial endotoxins and pro‐inflammatory metabolites, further exacerbating gut dysbiosis and inflammation. Previous studies have demonstrated that specific *Lactobacillus* strains can upregulate MUC2 gene expression, thereby enhancing epithelial barrier function, reinforcing mucosal defense, and reducing pathogen invasion (Breugelmans et al. 2022; Qin et al. 2022). *Lactobacillus* species are also known to stimulate mucin secretion and maintain tight junction integrity by modulating key signaling pathways, such as the PI3K/Akt and MAPK pathways, which are critical for epithelial homeostasis (Gao et al. 2023). The improvement in stool consistency observed in the LA85 intervention group in this study suggests that LA85 may contribute to strengthening the intestinal barrier, thereby mitigating the negative impact of antibiotic‐induced barrier dysfunction. Furthermore, research indicates that Lactobacillus strains can modulate the NF‐κB signaling pathway, which plays a central role in regulating inflammatory responses. LA85 supplementation may exert its protective effects by downregulating the expression of pro‐inflammatory cytokines such as TNF‐α, IL‐6, and IL‐1β while upregulating anti‐inflammatory cytokines such as IL‐10, thereby mitigating antibiotic‐induced mucosal inflammation and enhancing epithelial repair (Han et al. 2023). Notably, *Lactobacillus* strains have also been reported to influence T regulatory (Treg) cell differentiation, leading to immune tolerance and reduced inflammatory responses, which may further contribute to their protective role in AAD (Ness et al. 2021). A previous study using a murine UC model further indicated that LA85 effectively reduced pro‐inflammatory cytokine levels and alleviated intestinal inflammation, highlighting its broader anti‐inflammatory properties (Han et al. 2023). Given the similarities between antibiotic‐induced dysbiosis and inflammatory bowel disease pathophysiology, these findings suggest that LA85 may not only protect against AAD but may also have therapeutic potential in other conditions involving gut barrier dysfunction and chronic inflammation. Compared to prior studies, this research provides additional clinical evidence supporting the potential role of LA85 in AAD prevention. However, further investigation is required to elucidate the key molecular pathways underlying its effects. Future studies should incorporate cytokine profiling, histological analysis, and gut permeability assessments to further characterize LA85's role in intestinal barrier integrity. Additionally, integrating multi‐omics approaches, including transcriptomics and proteomics, may provide deeper insights into the strain‐specific regulatory mechanisms governing epithelial protection and immune modulation.

Although this study provides preliminary evidence supporting the use of LA85 for AAD prevention, certain limitations must be acknowledged. Firstly, the relatively small sample size may have limited statistical power, potentially obscuring significant differences in some efficacy parameters. Secondly, despite randomization, a modest baseline imbalance in age was observed between groups. While the clinical impact of this difference is likely limited, future studies should consider stratified randomization or covariate adjustment to minimize such imbalances. Thirdly, all authors are affiliated with the company that developed and holds the patent for LA85. While the study was conducted in accordance with ethical standards and transparency was maintained throughout, this potential conflict of interest should be considered when interpreting the results. Additionally, the short follow‐up duration precluded an assessment of the long‐term impact of LA85 on gut microbiota recovery. The absence of microbiome analysis further constrains mechanistic insights. Future studies should incorporate larger sample sizes and extended follow‐up periods, leveraging metagenomic and metabolomic approaches to comprehensively investigate the effects of LA85 on gut microbiota composition and functionality. Moreover, evaluating the efficacy and safety of LA85 supplementation in diverse populations, including immunocompromised individuals and elderly patients, will be essential for broader clinical applicability. The findings of this study lay a foundation for the clinical application of LA85 in AAD prevention, and future research will be instrumental in further elucidating its clinical benefits and mechanisms of action.

## 5 Conclusions

This study provides compelling evidence supporting the potential role of *Lactobacillus acidophilus* LA85 in preventing AAD. The randomized, double‐blind, placebo‐controlled trial demonstrated a trend toward reduced diarrhea incidence, shortened diarrhea duration, improved stool consistency, and enhanced gastrointestinal quality of life, likely through mechanisms involving pathogen inhibition, gut microbiota modulation, intestinal barrier enhancement, and immunoregulation. While the findings align with prior research on Lactobacillus strains, further investigations are needed to elucidate strain‐specific mechanisms, particularly regarding age‐dependent efficacy and long‐term microbiota modulation. Despite the study's limitations, including a relatively small sample size, short follow‐up period, and lack of direct microbiome analysis, these results provide a foundation for future research integrating multi‐omics approaches to validate LA85's effects. Expanding its clinical evaluation across diverse populations, particularly immunocompromised individuals and the elderly, will be essential to establishing its broader applicability in mitigating antibiotic‐induced gastrointestinal disturbances.

## Author Contributions

**Jianguo Zhu:** conceptualization, methodology, investigation, data curation, formal analysis, funding acquisition, software, writing – original draft. **Yukun Sun:** data curation, formal analysis, software, project administration, validation, visualization. **Yunjiao Zhao:** investigation, methodology. **Yao Dong:** investigation, methodology. **Zhonghui Gai:** writing – review and editing. **Shuguang Fang:** supervision, methodology, investigation.

## Ethics Statement

This study was conducted in accordance with the Declaration of Helsinki and its subsequent revisions. The protocol was reviewed and approved by the Clinical Research Ethical Committee Parc de Salut Mar (Barcelona, Spain) (study code: MTH_WEC0220).

## Consent

Written informed consent was obtained from all participants prior to enrollment in the study.

## Conflicts of Interest

All authors are employees of the company that developed and holds proprietary rights to *Lactobacillus acidophilus* LA85. While the study was conducted in accordance with internationally accepted ethical standards, and all data were collected and analyzed objectively, this affiliation may represent a potential competing interest. The authors affirm that they have disclosed all relevant relationships and that these affiliations did not influence the scientific integrity of the research or the conclusions drawn.

## Data Availability

The data presented in this study are available within the article.
